# Herpes Zoster Induced by the Shingrix Vaccine: A Case Report and Literature Review

**DOI:** 10.7759/cureus.102575

**Published:** 2026-01-29

**Authors:** Kristin Satow, Erin L Sherer, Anastasia Orlandi, Emma DeBaene

**Affiliations:** 1 Health Unit, U.S. Department of State, Washington, D.C., USA; 2 Medicine, Michigan State University, East Lansing, USA; 3 Human Biology, Michigan State University, East Lansing, USA; 4 Molecular and Cellular Biology, University of Illinois, Urbana-Champaign, USA

**Keywords:** herpes zoster virus, immunocompromised, shingles, varicella, zoster vaccine

## Abstract

Herpes zoster (HZ) is a dermatological condition that is caused by the reactivation of the varicella zoster virus (VZV). The recombinant zoster vaccine (RZV; Shingrix, GlaxoSmithKline Biologicals, Rixensart, Belgium) is approved in the United States for the prevention of HZ reactivation in adults aged >50 years. We present a case of a 50-year-old immunocompetent female patient who developed HZ within the first 72 hours post Shingrix vaccination. The patient was evaluated by their primary care provider for a painful, vesicular rash, along with headache and fatigue. A diagnosis of Shingrix vaccine-induced HZ was suspected. A 10-day course of the antiviral acyclovir was initiated. The rash improved after 21 days but then reemerged, and the patient was then given another course of acyclovir. The patient developed post-herpetic neuralgia (PHN) that persisted for several months following resolution of the skin rash. The patient did not receive the second dose of the vaccine. This case highlights a rare adverse effect observed in a patient who received the Shingrix vaccine. The diagnosis of HZ is largely clinical, and therefore vaccine-induced HZ should be suspected when no other cause can be identified. While further studies into vaccine components or patient risk factors that may trigger HZ infection after vaccination are necessary to reduce the development of HZ in the post-vaccination period, the benefits of vaccination still outweigh the risk.

## Introduction

The varicella zoster virus, also known as VZV, is the main cause of both the chickenpox and herpes zoster (HZ) diagnoses. Known as an alpha herpesvirus, its genes first affect the cells of the upper respiratory tract and mucosa, leading to a primary infection of chickenpox, most often seen in children [1]. This mechanism involves VZV cells reactivating and traveling through the lymph nodes to cause a configuration change in the T-cells that carry them, forcing the T-cells to act against the body's immune system [2]. After the primary infection has resolved, VZV establishes latency in the dorsal root ganglia and may reactivate (often decades later) to cause a secondary infection called HZ, or shingles. During reactivation, the virus replicates within the neurons and travels along nerves to the skin [3]. VZV evades the immune system by suppressing interferon signaling and MHC Class II antigen presentation, which delays host immune recognition and helps it persist [3]. As a result, patients may experience a multitude of symptoms, including cutaneous rash and postherpetic neuralgia (PHN), often lasting for months after infection. With PHN, patients may experience sensitivity, pain, and numbness throughout the whole body from the death of sensory nerves [4].

To protect against the detrimental effects of HZ, it is important to get vaccinated. In the United States, the recombinant zoster vaccine (RZV; Shingrix, GlaxoSmithKline Biologicals, Rixensart, Belgium) has been approved to be given intramuscularly to prevent HZ in adults aged >50 years [5]. Shingrix differs from the earlier live-attenuated zoster vaccine (ZVL; Zostavax, Merck & Co., Inc., Whitehouse Station, NJ, USA) in both composition and immunologic profile. Shingrix is a non-live, subunit vaccine that contains recombinant glycoprotein E (gE) and an adjuvant system, administered in a two-dose schedule. In contrast, Zostavax is a single-dose, live-attenuated formulation derived from the Oka strain of VZV. Because Shingrix is non-replicating, it can be safely administered to immunocompromised individuals, a major advantage over Zostavax, which is contraindicated in these populations. Shingrix has been found to be 97.2% effective at preventing HZ infection [5]. While Shingrix is highly efficacious at preventing HZ infection, it does have side effects, and a recent large-scale study observed a transient increase in shingles presentations in adults aged ≥65 years within 21 days following dose 1 [6]. This increase was not seen after dose 2 or in younger adults and was absent in hospital data, suggesting mild, self-limited cases. The hypothesized mechanism for developing HZ infection after vaccination involves the vaccine’s AS01B adjuvant, which stimulates a strong innate immune response. In older adults with immunosenescence or compromised immunity, this may briefly suppress immune surveillance of latent VZV, enabling a short-term opportunity for reactivation [6].

## Case presentation

A 50-year-old female patient presented to her primary care provider with a one-day history of rash and neck pain, four days after receiving the Shingrix vaccine. Prior to noticing any skin changes, the patient experienced headache and burning pain on the right side of her neck and thought her necklace was the potential cause. However, within 24 hours of the initial pain, the patient reported other symptoms, including a headache, fatigue, and a rash. The rash was localized to the right neck and had not spread anywhere else or crossed the midline. The patient denied facial or ocular rash, ear pain, itching, drainage, open wounds, recent illnesses, and use of new topical skin products on the affected area.

The patient’s past medical history included chickenpox infection as a child. The patient denied any drug allergies. Her surgical history included remote back and knee surgeries. The patient reported mild stress levels related to work and family responsibilities, but indicated that it felt manageable and that there were no acute stressors.

On examination, the patient was alert, oriented, and pleasant. The patient did not present with any signs of distress. The patient was afebrile, and other vital signs were within normal limits.

The patient’s cardiac and respiratory examinations were within normal limits.

**Figure 1:**
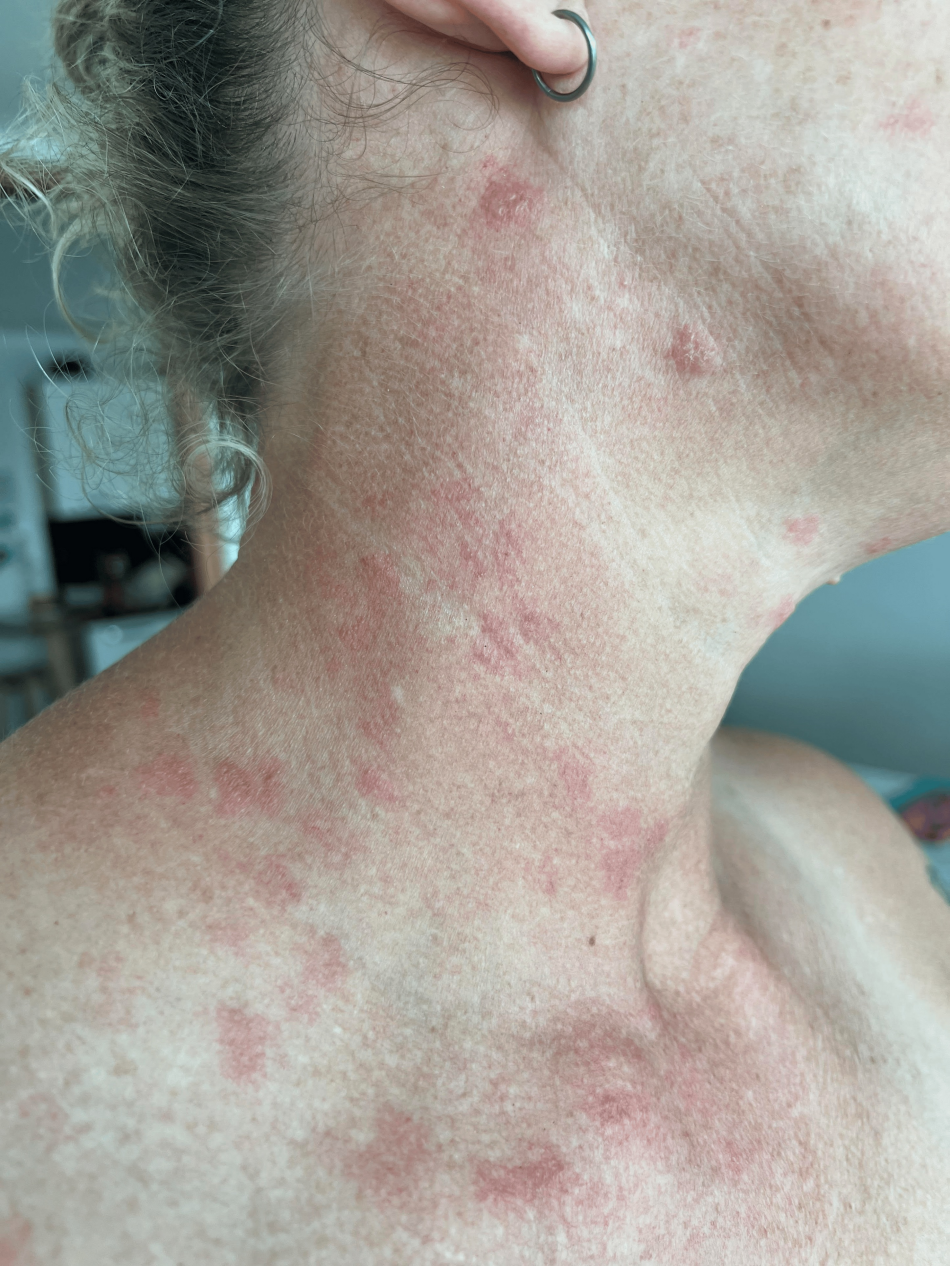
Image of the patient with the rash approximately 72 hours after receiving the Shingrix vaccine.

**Figure 2:**
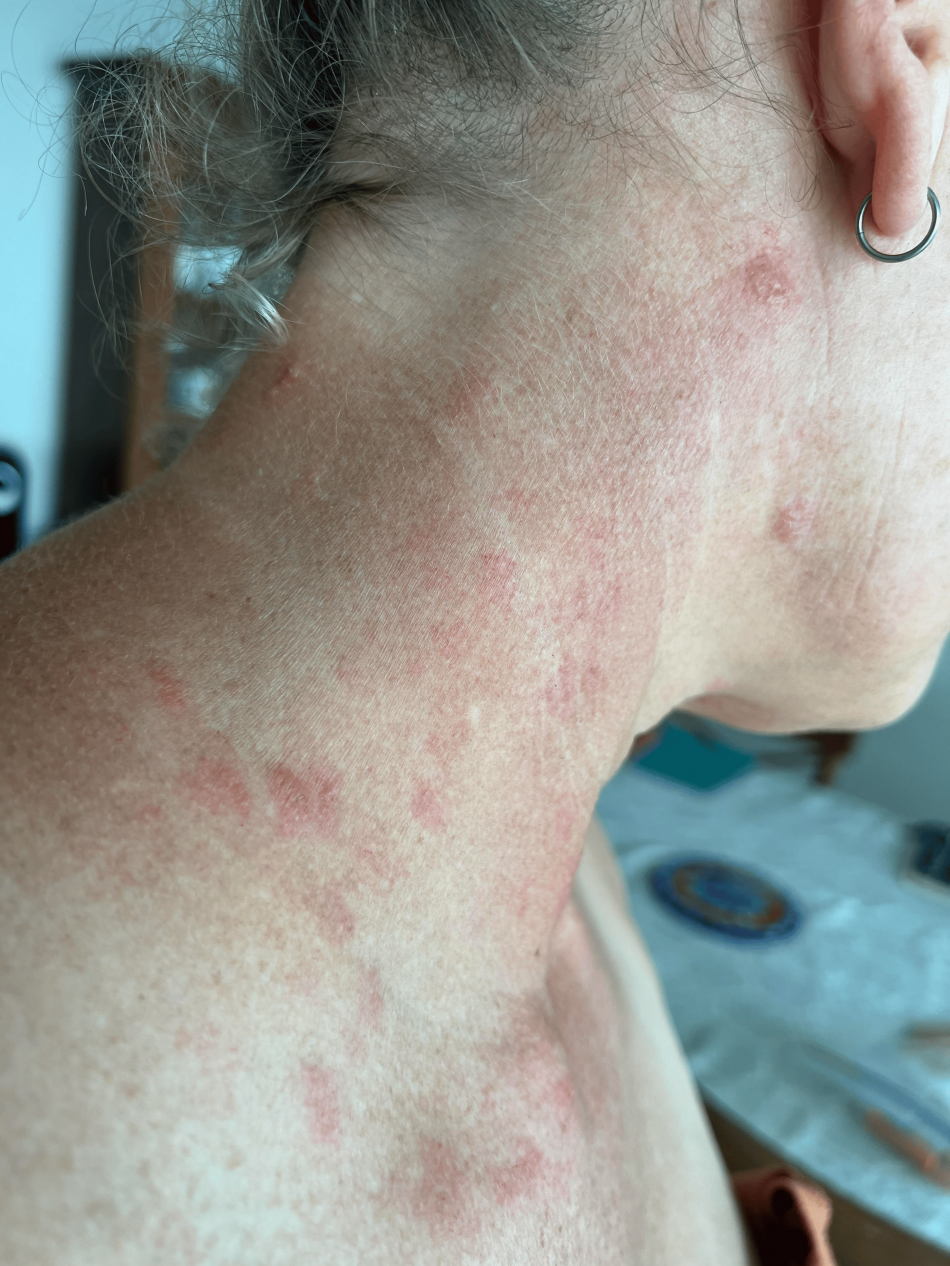
Image of the patient with the rash approximately 72 hours after receiving the Shingrix vaccine.

The patient’s dermatologic examination was notable for multiple vesicular lesions over the right supraclavicular area in the C3-C5 dermatomes (Figures 1, 2). The ocular and oropharynx were clear. Visual inspection of the ear and otoscopic examination revealed normal findings.

Differential diagnosis

Shingles is primarily a clinical diagnosis based on history and physical examination findings [7]. In the present case, the diagnosis of HZ was made based on the findings of localized dermatomal rash, unilateral distribution, and vesicular lesions. Although no virological confirmation (VZV polymerase chain reaction or direct fluorescent antibody testing) was obtained, this approach to diagnosis aligns with routine clinical practice [7].

The differential diagnoses that were considered and excluded were: contact dermatitis (poorly demarcated, lacks neuropathic pain), impetigo (may have vesicles, but not typically dermatomal and more frequent in children), and insect bites or cellulitis (generally not vesicular and does not include neuropathic pain).

Treatment

The patient was started on oral medication acyclovir 800mg every five hours for suspected HZ infection for 10 days. The patient was also given ibuprofen and acetaminophen as needed (prn) and gabapentin (dose range of 300mg to 600mg every eight hours) for pain control. When this was insufficient for pain management, the patient was also started on amitriptyline 10mg every night as needed.

Outcome and follow-up

**Figure 3:**
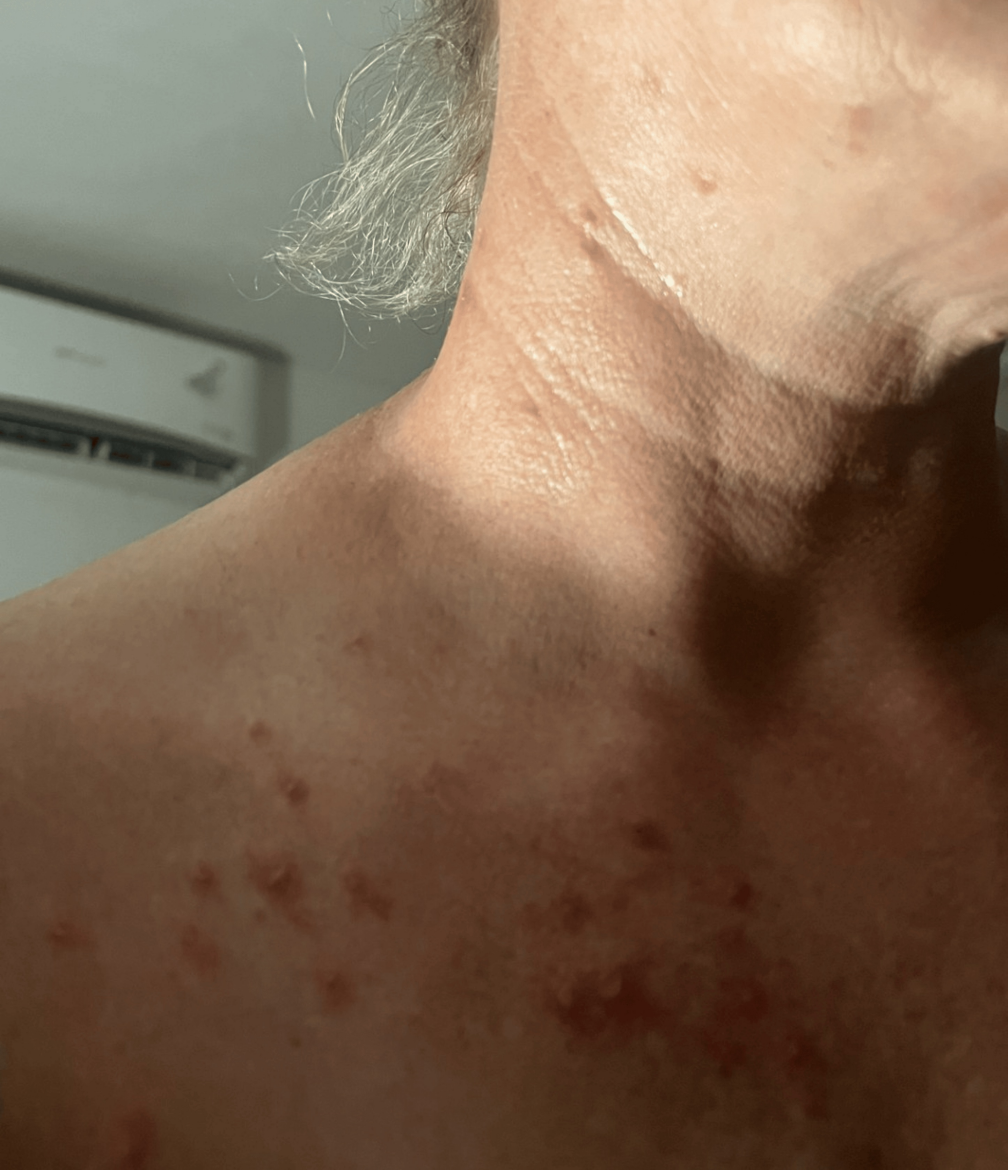
Anterior image of the patient with the rash approximately six weeks after receiving the Shingrix vaccine.

**Figure 4:**
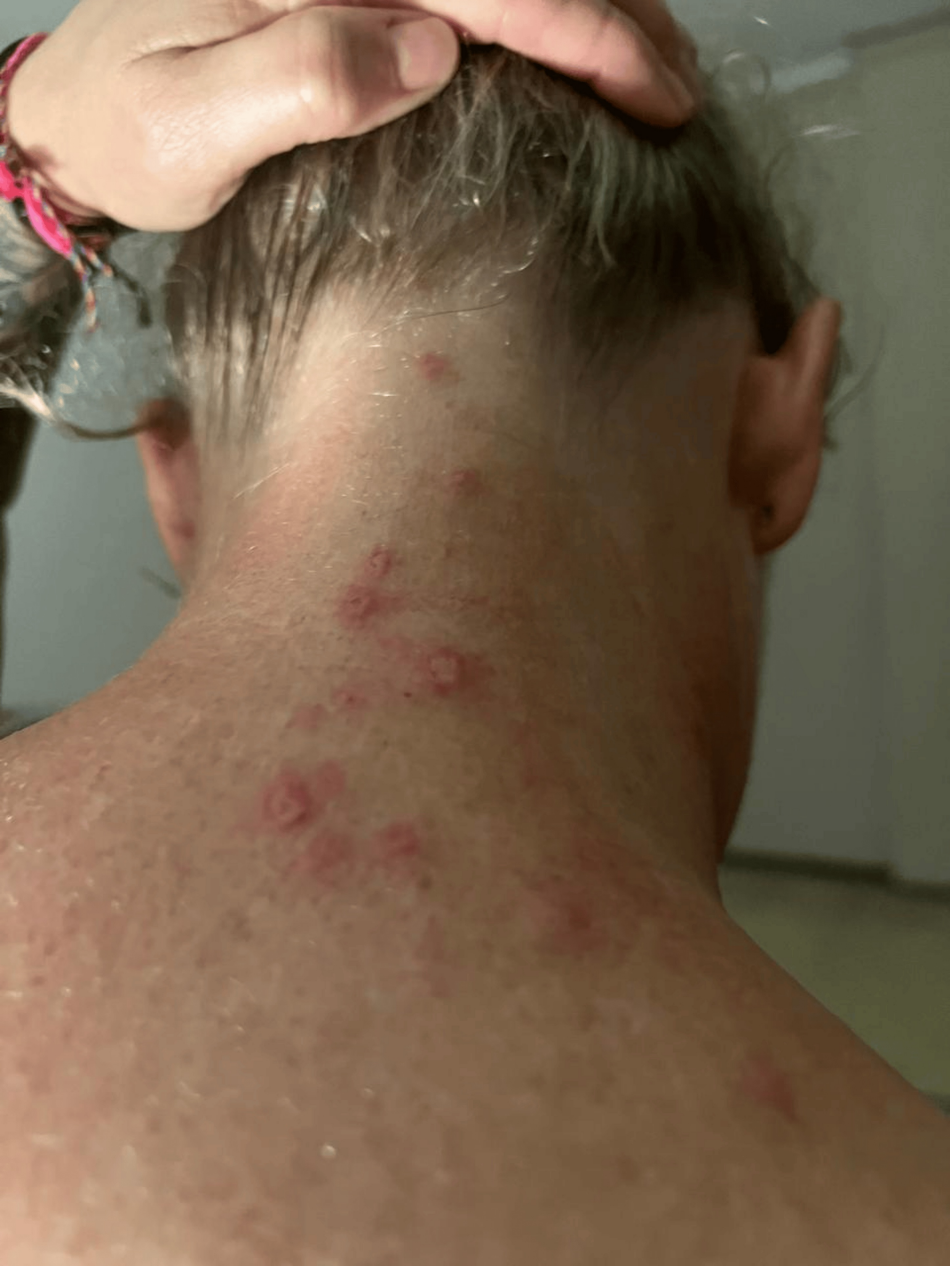
Posterior image of the patient with the rash approximately six weeks after receiving the Shingrix vaccine.

The patient continued to have the rash for approximately 21 days. The rash reemerged (Figures 3-4) six weeks after the onset of symptoms and four weeks after the complete resolution of lesions. The patient developed right ear itchiness, without lesions at this point, and the lesions resolved after another course of 10 days of acyclovir. The patient also continued to have PHN for several months following the diagnosis of HZ. The patient did not receive the second Shingrix dose on schedule.

## Discussion

A comprehensive literature review was conducted to identify cases of vaccine-induced HZ.

As in the case of our patient, HZ typically develops unilaterally in a dermatomal distribution (Figure 4). Although HZ may occur in the absence of a rash, the most common presentation is the appearance of a unilateral, dermatomal rash that is painful, pruritic, or both [8]. The dermatomes most commonly affected by HZ are the thoracic, followed by the cervical and trigeminal dermatomes [8].

**Figure 5:**
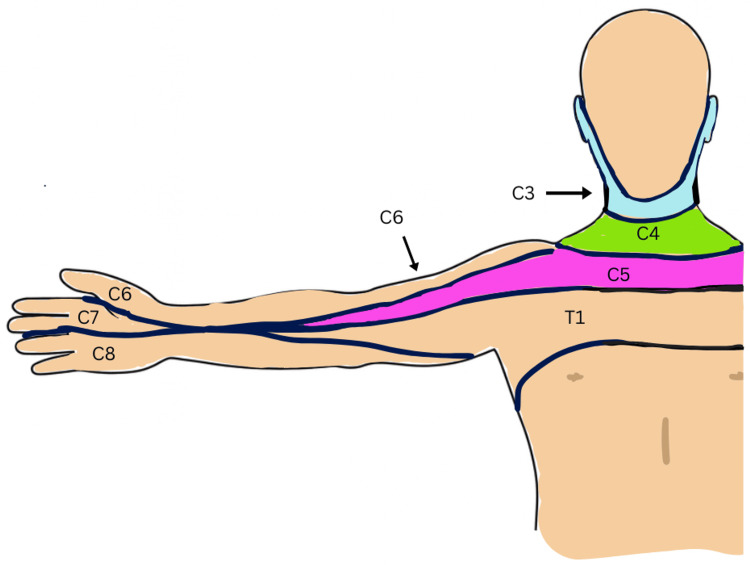
Distribution of herpes zoster rash in the patient was located in the C3, C4, and C5 dermatomes (as noted in blue, green, and pink). This figure has been created by the authors.

While side effects listed on the Shingrix website do not include HZ reactivation, there have been case reports of skin rashes consistent with HZ occurring shortly after vaccination [6,9-11]. Most people experience only mild vaccine reactions such as soreness at the injection site, mild fever, or fatigue [11]. However, less common immune-related or neurologic events such as Guillain-Barré syndrome (GBS) and lupus have been highlighted in case reports [12,13].

A few case reports have highlighted HZ occurrences after Shingrix vaccination. One case in the United States described a 73-year-old immunocompetent female who presented with a painful, itchy dermatomal rash three days after receiving the Shingrix vaccine [9]. Another case in the United States reported a 32-year-old immunosuppressed female who presented with a multidermatomal itchy rash over the vaccinated arm within 24 hours of receiving the first dose of the Shingrix vaccine [10]. A 60-year-old immunocompromised female developed a painful, itchy rash within one week of the first dose of the Shingrix vaccine [14]. Additionally, a large case-series analysis from Australia found an increase in HZ diagnoses during the first three weeks after the initial vaccine dose, with the highest incidence occurring within one to seven days, among adults aged 65 and older [6]. Interestingly, the analysis found that the increase in diagnosis did not continue after the second dose, and that individuals who completed both doses of the vaccine had a significantly lower overall risk of developing HZ in the future [6].

**Table 1:** Summary of case reports of HZ infection after the RZV vaccine HZ: herpes zoster; RZV: recombinant zoster vaccine; VZV: varicella zoster virus; PCR: polymerase chain reaction

Study	Population	Timing of Onset	Confirmation Method	Comments
Shetty et al. (2025) [6]	Adults >65-years-old (n=7,189)	Higher incidence within one to seven days after the first vaccine dose	Clinical	Transient risk increase in HZ after first RZV vaccine dose; no increase after second dose
Mittal et al. (2022) [9]	73-year-old female with thoracic dermatomal rash	Within three days after the first vaccine dose	Clinical	Patient received influenza vaccine at the same time as RZV; authors acknowledged previous Zostavax administration (10 years prior) may have also played a role; authors acknowledged uncertainty around causality
Housel & McClenathan (2020) [10]	32-year-old immunocompromised female with multidermatomal itchy rash	Within 24-hours of the first vaccine dose	PCR was positive for VZV; skin biopsy also confirmed the diagnosis	First published post-RZV zoster case report; atypical rash distribution; authors considered immune-mediated reaction and acknowledged uncertainty around causality
Altukhaim et al. (2023) [14]	60-year-old immunocompromised female with a painful, itchy rash	Within one week of the first vaccine dose	Clinical	Case suggests possible vaccine-triggered reaction in an at-risk patient; authors acknowledged uncertainty around causality

While the patient in our case report was immunocompetent and only 50 years old, there are risk factors that could have contributed to HZ reactivation. Research indicates that gender impacts the risk of HZ reactivation, with females having a higher risk than males [15]. Additionally, states of psychological stress can increase the risk of HZ reactivation [3]. Other risk factors include increased age, decreased immunity, acute infection, depression, and chronic diseases such as diabetes, cardiovascular disease, and many others [3].

The evidence supports Shingrix as a generally safe and highly protective vaccine, with most side effects being mild and temporary [5,8]. There is even emerging evidence that it may reduce the risk of developing dementia [16]. However, the small number of reports describing autoimmune reactions, GBS, or early post-dose-one reactivation highlights the importance of both patient and clinician education and awareness. As vaccination rates increase, these observations may help lead to discussions about benefits and risks and ensure that patients receive clear and balanced information.

## Conclusions

Vaccine-induced HZ is difficult to evaluate because proving causality is nearly impossible. While the temporal proximity of our case raises the possibility of a vaccine-related event, no definitive causal relationship can be established without objective confirmation. The diagnosis was made clinically, in line with routine practice, and no laboratory testing was performed. Although the patient did experience a suspected case of HZ with PHN, the symptoms resolved with medical management and time. By publishing this case report, we hope to add to the limited body of literature on this topic, ultimately helping clinicians in identifying and managing similar post-vaccination reactions. Despite this patient’s adverse event, this case study should not discourage providers from administering the Shingrix vaccine to reduce the incidence and complications of HZ in patients who meet the eligibility criteria.
